# A non-canonical androgen signaling pathway drives microglial activation and tau pathology in females

**DOI:** 10.21203/rs.3.rs-10286730/v1

**Published:** 2026-07-16

**Authors:** Xu Chen, Daniel Munoz-Mayorga, Xinchen Lyu, Suborno Jati, Jibin Zhang, Leigh-Ana Rossitto, Yuren Tao, Shanshan Zhao, Enfu Hui, Alexander Kauffman, Bokai Zhu

**Affiliations:** University of California, San Diego; University of California San Diego; University of California San Diego; University of California San Diego; University of California San Diego; University of California San Diego; University of California San Diego; University of California San Diego; University of California San Diego; University of California San Diego; University of Pittsburgh

## Abstract

Alzheimer’s disease (AD) disproportionately affects women, who exhibit greater vulnerability to Tau pathology and neuroinflammation. The precise mechanisms underlying this vulnerability remain elusive, although sex hormones are thought to play a pivotal role. Here, we report that supplementation with the non-aromatizable androgen dihydrotestosterone (DHT) exacerbates Tau pathology in female tauopathy models, with microglia as the main driver of this effect. DHT treatment upregulates proinflammatory gene expression in microglia and promotes the disease-associated microglia (DAM) phenotype in a Trem2-dependent manner. Surprisingly, these effects are independent of the canonical androgen receptor (AR) and instead depend on the orphan nuclear receptor TR4, which mediates DHT-driven effects by transcriptionally regulating *Trem2* in microglia. Moreover, TR4 protein levels are elevated in postmortem brain tissue from Braak stage 6 female AD patients and correlate with p-Tau levels. Together, our findings uncover a non-canonical DHT–TR4–*Trem2* signaling axis in microglia and identify TR4 as a key regulator of neuroinflammation in female neurodegeneration, providing mechanistic insight into female-specific vulnerability to AD.

## Introduction

Alzheimer’s disease (AD), the most common type of dementia, disproportionately affects women, who account for two-thirds of individuals living with the disease^1,2^. Pathologically, women on the AD continuum exhibit greater vulnerability to developing Tau pathology than men^3–6^, highlighting biological differences in disease progression that remain insufficiently characterized^7^. Recent research indicates that sex-specific microglial responses could significantly contribute to these divergent pathological outcomes^8–12^.

Microglia are increasingly recognized as central mediators of tau-driven neuroinflammation and neurodegeneration^13–15^. The microglial response to protein aggregates relies heavily on signaling via triggering receptor expressed on myeloid cells 2 (TREM2), a receptor that orchestrates disease-associated microglial activation and contributes to tau-associated neuroinflammation and neurodegeneration^16–18^. Notably, TREM2-mediated signaling in response to Tau pathology is sexually dimorphic, with females being more vulnerable to neurodegeneration and cognitive deficits^19,20^. Given the pivotal role of TREM2 in female Tau pathology, identifying upstream transcriptional mechanisms that regulate TREM2 may provide important insight into sex-specific vulnerability.

The decline in circulating sex hormones during menopause and the decrease in locally produced estrogen have profound effects on female brain aging, contributing to amyloid β deposition, tau pathophysiology, metabolic dysfunction, and dysfunctional inflammatory responses^21–25^. In contrast, the role of androgens in female brain physiology remains poorly understood, despite their importance as steroid hormones throughout life^26^. Androgen levels can also become elevated in conditions such as polycystic ovarian syndrome (PCOS)^27^ and during exogenous androgen therapy^28^, both of which are associated with profound metabolic and immune alterations^29–31^. These observations raise the possibility that androgen signaling regulates microglial function, thereby influencing neuroinflammatory responses and susceptibility to neurodegeneration. Whether androgen signaling regulates TREM2-dependent microglial responses during tauopathy, and if so, through what molecular mechanisms, remains unknown.

In this study, we demonstrate that high levels of 5α-dihydrotestosterone (DHT), a non-aromatizable androgen, exacerbate Tau pathology by promoting Trem2-dependent microglial inflammatory responses. Mechanistically, we identify the orphan nuclear receptor TR4 as a non-canonical androgen-responsive transcriptional factor that directly regulates *Trem2* expression, thereby driving microglial activation and accelerating Tau pathology in the female brain. Furthermore, we show that TR4 expression is elevated in female AD brains and positively correlates with Tau pathology, supporting the clinical relevance of this pathway. Overall, these findings establish a non-canonical DHT–TR4–TREM2 signaling axis that links androgen signaling to microglia activation and Tau pathogenesis, providing a new mechanistic framework for understanding female-specific vulnerability to AD.

## Results

### DHT supplementation exacerbates Tau pathology and neural injury in female P301S mice

**Figure 1:**
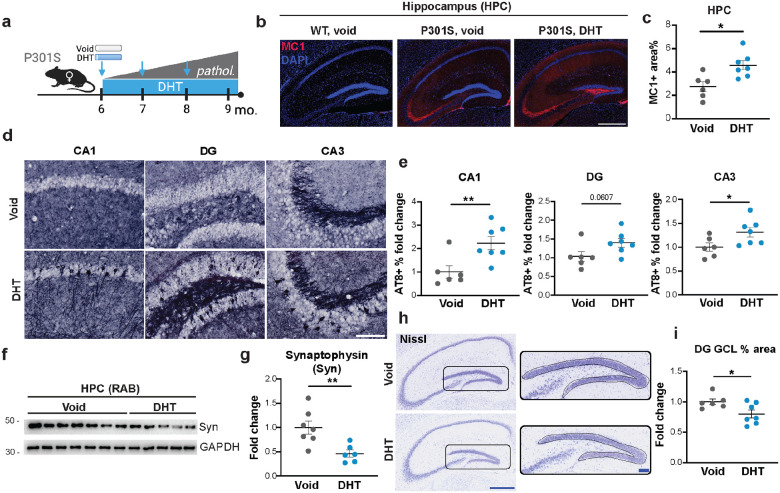
DHT supplementation exacerbates Tau pathology and neurodegeneration in female P301S mice. **a** A schematic of the DHT supplementation paradigm in female P301S mice (Created in BioRender). **b, c** MC1 immunostaining of the hippocampi (HPC) of female WT and P301S mice showing higher MC1+% immunoreactive area in DHT-treated females, quantified in (**c**). n = 6 void, n = 7 DHT. Scale bar: 500 μm. **d, e** AT8 immunostaining (DAB) images of hippocampal regions showing increased p-Tau and NFT-like structures in DHT-treated females, quantified in (**e**). n = 6 void, n = 7 DHT. Scale bar: 100 μm. **f, g** Immunoblot showing a decrease in synaptophysin (Syn) levels in RAB-soluble hippocampal lysate from DHT-treated females, quantified in (**g**). n = 7 for void, n = 6 for DHT. **h, i** Nissl staining showing a smaller area of the DG granule cell layer (GCL) in DHT-treated females, quantified in (**i**). n = 6 void, n = 7 DHT. Scale bar: 100 μm. **c, e, g, i** Values are mean ± SEM. *p<0.05, **p<0.01; numeric values non-significant by Welch’s t-test.

To determine the effects of elevated androgen levels on Tau pathogenesis in females, we supplemented adult female P301S human Tau transgenic mice (P301S)^32^ with DHT, a non-aromatizable androgen^33^. Unlike testosterone, which can be converted into estrogen by aromatase, DHT cannot be aromatized into estrogen^34^, allowing us to separate androgen-specific effects. We treated P301S female mice at 6 months of age with a 10-mm DHT-packed SILASTIC tubing or a void implant^35^ for 12 weeks, resulting in increased cortical androgen receptor (AR) levels, confirming DHT-AR engagement (Fig. 1a; **Supplementary Fig. 1a, b**). Immunohistochemistry (IHC) showed elevated Tau aggregates (marked by the conformation-specific MC-1 antibody) and increased Tau phosphorylation (by anti-pTauS202/T205, AT8) (Fig. 1b–e). Notably, diaminobenzidine (DAB) AT8 immunoreactivity was particularly evident in structures morphologically consistent with neurofibrillary tangles (NFTs) (Fig. 1d). Importantly, we also detected lower synaptophysin protein levels in hippocampal lysates (Fig. 1f, g) and decreased area of the granule cell layer (GCL) at the dentate gyrus (DG), as assessed by Nissl staining (Fig. 1h, i) in DHT-treated P301S females. These findings suggest that high levels of DHT induce Tau pathology and neural injury in female tauopathy brains.

To elucidate whether these effects were driven by DHT exposure in brain tissue, we used female organotypic hippocampal slice cultures (OHSCs)^36–38^. Consistent with our *in vivo* observations, DHT-treated OHSCs transduced with AAV-hTau^P301S^ and inoculated with preformed Tau fibrils (PFFs, K18/PL) **(Supplementary Fig. 1c)** showed increased MC1+% area **(Supplementary Fig. 1d-e)**. Immunoblotting showed that p-Tau levels were unchanged in RIPA-soluble OHSC lysates. However, there was a trend toward reduced total soluble Tau levels, suggesting decreased Tau solubility, consistent with exacerbated MC1 + Tau pathology **(Supplementary Fig. 1f, g)**. Taken together, our data illustrate that DHT supplementation in females exacerbates Tau pathology and Tau-mediated neurodegeneration, at least in part, through brain-intrinsic mechanisms.

### DHT drives disease-associated microglial activation in female tauopathy

**Figure 2:**
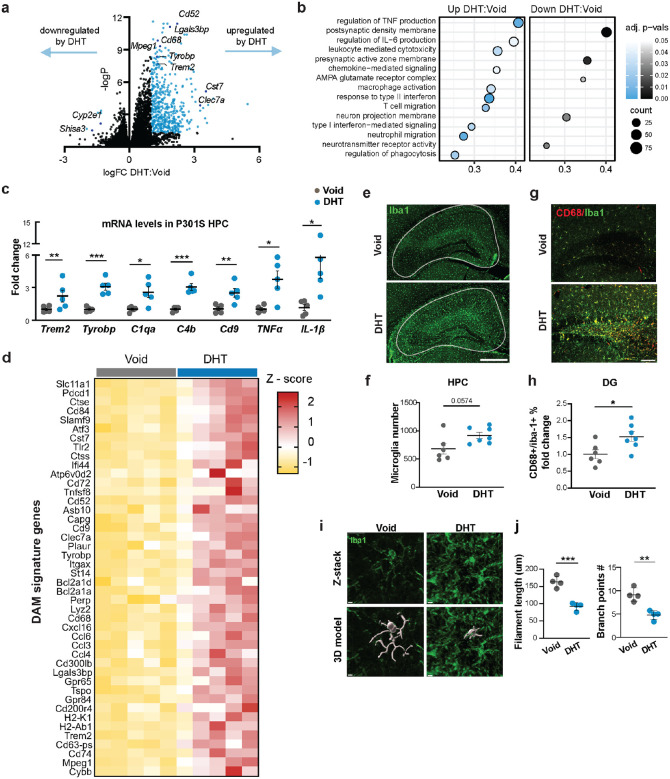
DHT drives disease-associated microglial activation in female tauopathy. **a** Volcano plot of differentially expressed genes (DEGs) in HPC of DHT-treated females compared to void. Genes with a fold change (FC) > 1 and with a p-adjusted value > 0.05 are highlighted in blue. **b** GSEA showing upregulation of inflammatory pathways and downregulation of synaptic membrane pathways in DHT-treated females. Input gene lists were ranked by score: −logP*FC. **c** RT-qPCR validation of selected significantly upregulated DEGs and inflammatory markers in HPC in DHT-treated P301S females. n = 5 per group. **d** Heatmap showing Z-scores of DAM genes among upregulated DEGs in P301S females. **e-h** Iba1 (e) and CD68 (g) immunofluorescent staining in whole HPC and DG showing increased reactive microglia in DHT-treated females, quantified in (**f, h**). n = 6 void, n = 7 DHT. Scale bar: 500 μm (**e**) and 100 μm (**g**). **i, j** Iba1 staining and 3D rendering by Imaris showing morphological changes in HPC suggestive of microglial activation in DHT-treated females, quantified in (**j**). n = 4 per group with the average of three fields analyzed by Imaris, scale bar: 20 μm. **c, f, h, j** Values are mean ± SEM. * p<0.05, **p<0.01, ***p<0.001: numeric values non-significant by Welch’s t-test.

To understand the mechanisms by which DHT exacerbated Tau pathology and neurodegeneration, we performed bulk RNA-seq on the hippocampi of female P301S mice treated with DHT. DHT supplementation induced mainly microglial activation genes (Fig. 2a), including *Trem2*, its adapter protein Dap12 (*Tyrobp*) and Trem2-dependent Disease Associated Microglia (DAM) DAM genes *Cst7* and *Clec7a*^16,17,39^ (Fig. 2a). Gene Set Enrichment Analysis (GSEA) further showed that DHT-upregulated genes were enriched for inflammatory pathways, including TNFα and IL-6 production (Fig. 2b). On the other hand, DHT-downregulated genes were enriched in pathways related to synaptic components (Fig. 2b), consistent with our pathological observations (Fig. 1f–i). RT-qPCR validated the top hits for microglial activation genes identified by bulk RNA-seq, as well as *TNFα* and *IL-1β*, confirming a robust pro-inflammatory state in DHT-treated females. (Fig. 2c). Consistently, DHT-treated females showed higher expression of DAM-associated gene signatures^16^ (Fig. 2d). These transcriptomic signatures were reflected at the protein level in DHT-treated females, as shown by Iba1/CD68 co-staining. Compared to the void-treated group, DHT-treated females exhibited a trend of increase in microglial density (Fig. 2e, f), and significantly increased CD68 + microglia (CD68+/Iba1+) in the dentate gyrus (DG) (Fig. 2g, h). Notably, Imaris 3D analysis confirmed that microglia in DHT-treated females exhibited reduced process complexity compared to void-treated females (Fig. 2i, j), consistent with the DAM-like phenotype commonly observed in neurodegenerative conditions, including tauopathies^16,40^. Overall, our findings indicate that high levels of DHT exacerbate microglial proinflammatory phenotype in female tauopathy brains.

### Trem2 mediates DHT-induced microglial dysfunction and Tau pathology

**Figure 3:**
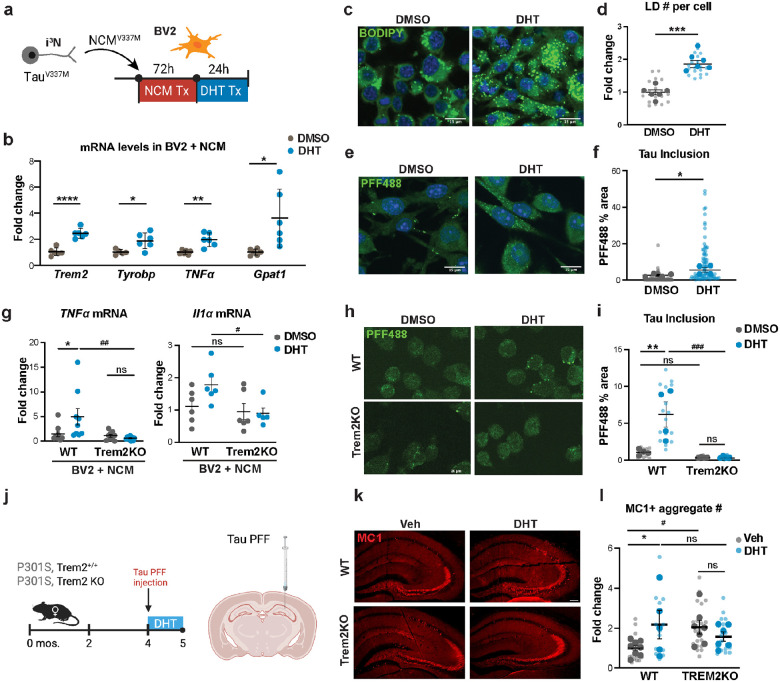
DHT triggers neuroinflammation in a cell-autonomous and Trem2-dependent manner. **a** Schematic of DHT treatment in NCM^V337M^-treated BV2 cells (Created by BioRender). **b** RT-qPCR showing DHT treatment (10 nm) increases the expression of *TNFα*, *Trem2*, *Tyrobp*, and *Gpat1* in NCM^V337M^-treated BV2 cells, n = 6 wells from two independent experiments. **c, d** BODIPY staining showing increased lipid droplets (LDs) in DHT-treated BV2 cells, quantified in (**d**), n = 6 coverslips/group, from three independent experiments. Translucent dots represent individual ROIs, and solid dots represent the average of individual coverslips (n=2–4 ROIs per coverslip). **e, f** Fluorescent images of BV2 cells treated with PFF488, showing increased Tau PFF inclusions in DHT-treated cells, quantified in (**f**). n = 4 coverslips/group from two independent experiments. Translucent dots represent individual cells, and solid dots represent the average of individual coverslips (n = 20 cells/coverslip). **g** RT-qPCR showing decreased cytokine expression of *TNFα* and *IL-1α* in Trem2KO cells upon DHT treatment, n = 9 wells from 3 independent experiments for *TNFα*, n = 6 wells from two independent experiments for *IL-1α*. **h, I** Fluorescent images of WT and Trem2KO BV2 cells treated with PFF488 concomitantly with vehicle or DHT, quantified in (**i**). n = 4 coverslips from two independent experiments. Each coverslip represents the average of 4 ROIs. Translucent dots in the bar graph represents the average fluorescent intensity per ROI. Each solid dot in the bar graph represents the average fluorescent intensity per coverslip. Scale bar: 20 μm. **j** Schematic of PFF injection and DHT treatment in P301S and P301S/Trem2KO mice. **k, l** Immunostaining of MC1+ Tau aggregates showing an increase in DHT-treated P301S mice and void implanted P301S/Trem2KO, quantified in **(l)**. Void; P301S (n = 8) mice, DHT; P301S (n = 5) mice, Void; P301S/Trem2KO (n = 7) mice, and DHT; P301S/Trem2KO (n = 6) mice. Each translucent dot represents an individual slice, and each solid dot represents an individual mouse; three sections per mouse. Scale bar: 500 μm. **b** Values are mean ± SEM, *p<0.05, **p<0.01, ****p<0.0001 by Welch’s t-test. **g** Values are mean ± SEM, *,^#^p<0.05, **,^##^p<0.01, ns (non-significant) by 2-way ANOVA with Šídák’s multiple comparisons test. **d, f** Values are mean ± SEM of individual wells,*, ^#^p<0.05, **,^##^p<0.01, ***,^###^p<0.01; non-significant ns using a linear mixed-effect model (LMM) and **i, l** using a multilevel linear mixed-effect model (LMM); * and ^#^ indicate pairwise comparison between different factors.

The pronounced microglial activation induced by DHT led us to investigate whether DHT induces microglial inflammation and dysfunction in a cell-autonomous manner. We previously reported that incubation with neuronal conditioned media from iPSC-derived neurons carrying the V337M Tau mutation (NCM^V337M^) triggers a pro-inflammatory response and lipid droplet (LD) accumulation in BV2 cells (a female-derived microglial cell line)^41^. BV2 cells incubated with NCM^V337M^ followed by DHT treatment (Fig. 3a) exhibited increased expression of *Trem2*, *Tyrobp*, and *TNFα* as detected by RT-qPCR (Fig. 3b). DHT treatment also significantly increased the number of LDs in BV2 cells, corroborated by increased transcription of *Gpat1*, an LD-associated gene (Fig. 3b–d). Additionally, when loaded with fluorophore-tagged full-length Tau PFFs (PFF488), BV2 cells accumulated more PFFs after DHT treatment (Fig. 3e, f). Together, these findings suggest that DHT acts directly on microglia to induce inflammation and dysfunction in a tauopathy context.

Across both *in vivo* and *in vitro* experimental models, *Trem2* consistently emerged as a highly upregulated transcript following DHT treatment. Given its well-established role in orchestrating microglial function^16,17,39^, we investigated the extent to which Trem2 mediates these DHT-induced microglial responses. CRISPR-mediated *Trem2* KO in BV2 cells prevented DHT-induced upregulation of pro-inflammatory cytokines *TNFα* and *IL-1α* after NCM^V337M^ exposure (Fig. 3g). Furthermore, the absence of *Trem2* significantly reduced the amount of Tau PFF488 accumulated in BV2 cells after DHT and PFF co-treatment (Fig. 3h, i). To determine whether Trem2 also mediates the effects of DHT *in vivo*, we injected Tau PFFs into the hippocampus of P301S and P301S/Trem2KO mice, followed by 4 weeks of DHT supplementation (Fig. 3j). DHT supplementation increased hippocampal MC1 + tau aggregates in P301S mice whereas this effect was not observed in P301S/Trem2KO mice (Fig. 3k, l). Notably, void-implanted P301S/Trem2KO females exhibited a higher burden of insoluble Tau aggregates than the P301S controls, suggesting that while Trem2 limits Tau accumulation under basal conditions, it is also required for DHT-induced exacerbation of tau pathology (Fig. 3k, l). To isolate the impact of Trem2 on DHT-induced Tau pathology from peripheral influences, we derived OHSCs from female WT and Trem2KO mice. *Trem2* deletion prevented the DHT-induced increase in MC1 + aggregates in OHSCs **(Supplementary Fig. 2a, b)**. *Trem2* deficiency also attenuated the DHT-induced increase in CD68 protein levels **(Supplementary Fig. 2c, d)** and *TNFα* mRNA levels **(Supplementary Fig. 2e)**. Overall, these findings suggest a crucial role for *Trem2* in mediating the detrimental effects of DHT on Tau pathology in female tauopathy models, primarily through maladaptive inflammatory responses.

Because DHT classically signals through the AR, we next asked whether this canonical signaling pathway in microglia mediates these effects. However, AR mRNA expression in BV2 cells was undetectable by RT-PCR **(Supplementary Fig. 3a)**, consistent with a previous report^42^. Nonetheless, we generated microglia-specific AR-deficient OHSCs using *CX3CR1-CreERT2; AR*^*fl/fl*^ mice with tamoxifen-induced Cre expression, followed by DHT treatment **(Supplementary Fig. 3b)**. Despite efficient genomic recombination under Cre expression **(Supplementary Fig. 3c)**, microglial AR deletion failed to attenuate DHT-induced increases of p-Tau and MC1 + Tau aggregates **(Supplementary Fig. 3d-g)**. Together, these findings demonstrate that DHT cell-autonomously drives Trem2-dependent microglial dysfunction independently of canonical AR signaling, suggesting the existence of an alternative androgen-responsive regulatory mechanism.

### TR4 mediates androgen-induced microglial activation by transcriptionally regulating Trem2

**Figure 4:**
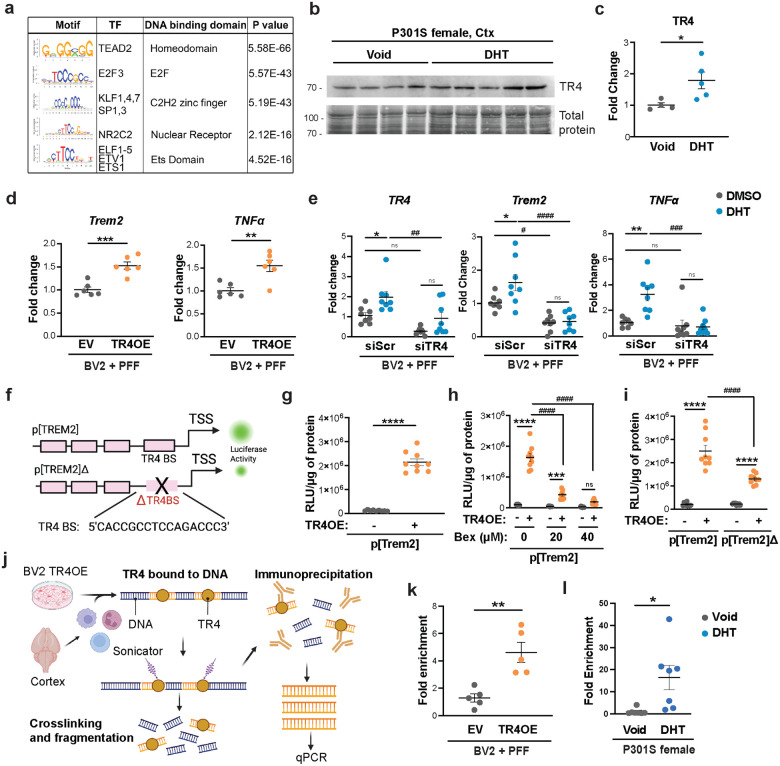
DHT regulates microglial transcription by activating TR4. **a** Motif enrichment analysis by SeqPos of significantly upregulated genes in DHT-treated females; TR4 (*Nr2c2*) emerged as one of the top hits. **b, c** Immunoblot showing an increase in TR4 levels in RIPA cortical lysates of DHT-treated P301S females, quantified in (**c**). n = 4 for void, n = 5 for DHT. **d** RT-qPCR showing increased expression of *Trem2* and *TNFα* in TR4OE BV2 cells. n = 6 wells from two independent experiments. **e** RT-qPCR showing *TR4* knockdown abolishes DHT-induced upregulation of *Trem2* and *TNFα*. *TR4* mRNA levels confirm knockdown efficiency. n = 8 wells from 3 independent experiments. **f** Schematic showing luciferase reporter design to assay human *TREM2* promoter activity in HEK293T cells (Created in BioRender). **g** Luciferase assay in HEK293T cells showing increased *TREM2* promoter activity in TR4OE cells, n = 9 wells/group from 3 independent experiments. **h** Luciferase assay in HEK293T cells showing dose-dependent suppression of *TREM2* transcriptional activity by bexarotene (Bex) treatment in TR4OE, n = 9 wells/group from three independent experiments. **i** Luciferase assay in HEK293T cells showing deletion of the top TR4 binding site (TR4BS) significantly reduced *TREM2* promoter activity in TR4OE cells. n = 9 wells/group from three independent experiments. **j** Schematic of the strategy for *in vitro* and *in vivo* ChIP to evaluate TR4 enrichment in the *Trem2* promoter (Created in BioRender). **k** ChIP-qPCR showing TR4 enrichment at the *Trem2* promoter in *TR4*OE BV2 cells, n = 5 wells/group from two independent experiments. **l** ChIP-qPCR showing TR4 enrichment at the *Trem2* promoter in the cortex of DHT-treated P301S females, n = 7 per group. **c, d, g, k, l** Values are mean ± SEM, *p<0.05, **p<0.01, ***p<0.001 from Welch’s t-test. **e, h, i** Values are mean ± SEM, *,^*#*^*p*<*0.05*, **,^##^p<0.01, ***,^###^p<0.001, ****,^####^p<0.0001, ns (non-significant) by 2-way ANOVA with Šídák’s multiple comparisons test; * and ^#^ indicate pairwise comparison between different factors

Given the lack of AR involvement in DHT-induced microglial activation and Tau pathology, we sought to uncover alternative regulators underlying DHT-induced transcriptional changes in females. Motif enrichment analysis and Landscape In Silico deletion Analysis (LISA) of significantly upregulated genes identified the nuclear receptor subfamily 2 group C member 2 (Nr2c2), also known as TR4, as a top candidate (Fig. 4a; **Supplementary Fig. 4a**). Since TR4 has previously been implicated in macrophage inflammatory activation and lipid metabolism^43–46^, we investigated whether it mediates the DHT-induced microglial phenotype. Consistent with computational predictions, immunoblot analysis revealed elevated TR4 protein levels in the cortex of P301S females treated with DHT (Fig. 4b, c). Normalized RNA-seq counts further showed upregulation of previously established TR4 transcriptional target genes (e.g., *Rela*, *Apoe*, and *Nfkb1*)^46,47^ in DHT-treated females **(Supplementary Fig. 4b)**. In addition, NCM^V337M^-treated BV2 cells showed elevated expression of *TR4* mRNA **(Supplementary Fig. 4c)**. These findings suggest that TR4 may be a key transcriptional mediator of DHT-induced microglial responses.

To determine whether TR4 functionally mediates DHT-induced inflammation and *Trem2* expression, we next performed gain- and loss-of-function experiments in BV2 cells. Overexpression of *TR4* (TR4OE) in BV2 cells treated with Tau PFFs led to increased *Trem2* and *TNFα* expression (Fig. 4d; **Supplementary Fig. 4d**), phenocopying DHT’s effects (Fig. 2c) and suggesting that TR4 gain-of-function is sufficient to induce an inflammatory microglial response. Conversely, siRNA-mediated knockdown of *TR4* abolished DHT-induced upregulation of *Trem2* and *TNFα* expression (Fig. 4e), suggesting that TR4 is necessary for DHT’s effects. Furthermore, Cellular Thermal Shift Assay (CETSA)^48^ in HEK293T cells overexpressing *TR4* **(Supplementary Fig. 4e)** revealed a rightward shift of the melting curve with DHT treatment compared to the vehicle, indicating that DHT increased thermal stability of TR4 **(Supplementary Fig. 4f, g)**, likely due to their biochemical interactions. These data support a model in which DHT interacts directly with TR4 and mediates DHT-induced transcriptional effects in microglia.

We next investigated whether TR4 directly binds to the *Trem2* promoter to regulate its transcription. We used Biomni AI^49^ to identify potential TR4 binding sites (TR4BS) within the human *TREM2* promoter **(Supplementary Fig. 4h)**. We designed a luciferase reporter construct containing four TR4BS most adjacent to the transcriptional start site (TSS) of the *TREM2* promoter (Fig. 4f). TR4 overexpression significantly increased *TREM2* promoter activity (Fig. 4g), which was dose-dependently reduced by bexarotene (Bex), a known antagonist of TR4^50,51^ (Fig. 4h). Furthermore, in line with the AI prediction, deletion of the strongest TR4BS reduced luciferase activity by approximately 50% (Fig. 4i). Finally, we sought to confirm the physical occupancy of TR4 at the Trem2 promoter via Chromatin Immunoprecipitation (ChIP)^52^ followed by qPCR (Fig. 4j; **Supplementary Fig. 4i**). Notably, both *TR4*OE BV2 cells and the cortex of DHT-treated female P301S mice showed a *Trem2* promoter region with enriched TR4 occupancy (Fig. 4k, l). Collectively, these findings suggest that DHT promotes TR4 binding to the *Trem2* promoter, thereby enhancing its transcriptional activity.

### TR4 is elevated in human Alzheimer’s disease and correlates with tau pathology

**Figure 5:**
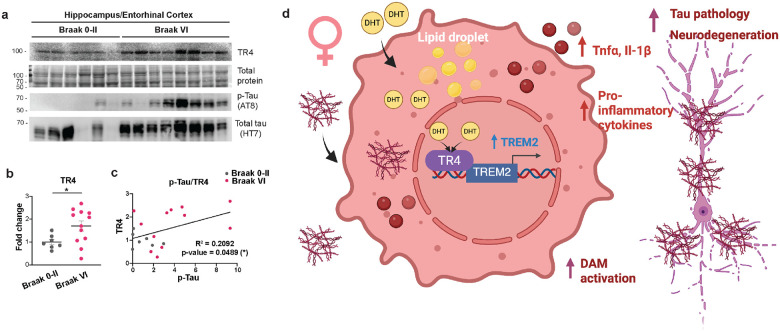
Increased TR4 levels in female AD brains support the clinical relevance of the TR4 hyperactivity in Tau pathogenesis. **a-c** Immunoblot analysis of postmortem brain lysates of female AD patients with TR4, p-Tau (AT8), and total Tau (HT7) antibodies, showing a significant increase in TR4 levels in Braak VI patients, quantified in (**b**), and a positive Pearson correlation between TR4 and p-Tau levels in (**c**). **b**, Values are mean ± SEM. *p<*0.05* by Welch’s t-test. **c**, Normalized values for TR4 and p-Tau were plotted, and a two-tailed Pearson correlation was used. **d** Schematic of the working model of the DHT-TR4-Trem2 axis regulating microglia activation in female tauopathy. DHT activates the nuclear receptor TR4 in female microglia, promoting TR4-dependent transcription of *Trem2*. Increased *Trem2* expression drives disease-associated microglial (DAM) gene expression, lipid droplet accumulation, and inflammatory cytokine production, thereby exacerbating Tau pathology and neurodegeneration.

Finally, to determine whether TR4 dysregulation extends to human AD/tauopathy, we obtained frozen autopsy specimens of the hippocampal/entorhinal area from female individuals with an AD diagnosis. Immunoblot of brain lysates showed that Braak stage VI patients had significantly higher TR4 protein levels compared to Braak 0-II patients (Fig. 5a, b). Furthermore, p-Tau levels were positively correlated with TR4 protein content at the individual patient level (Fig. 5c). Overall, our findings position TR4 as a novel microglial regulator in female AD Tau pathophysiology, which underlies DHT-induced exacerbation of microgliosis and tauopathy (Fig. 5d).

## Discussion

The molecular basis for women’s increased susceptibility to AD remains poorly understood^1^. In this study, we identify a previously unrecognized non-canonical androgen signaling pathway that promotes microglial activation and accelerates Tau pathology in female models of tauopathy. Through both *in vivo* and *ex vivo* experiments, we show that DHT exacerbates Tau pathology by inducing a DAM state through Trem2-dependent inflammatory responses, independently of the canonical AR-mediated pathway. Mechanistically, we identify the orphan nuclear receptor TR4 as a transcriptional regulator of Trem2 that links androgen exposure to maladaptive microglial activation. Together, these findings establish a DHT–TR4–Trem2 signaling axis that provides a mechanistic framework connecting hyperandrogenic states to female vulnerability in tauopathy.

Our findings build upon a growing body of evidence suggesting that Trem2-dependent microglial responses play a prominent role in neuroinflammation and the development of Tau pathology in females^7,9–12,19,20,53^. Previous studies have shown that the AD-associated R47H-TREM2 variant exacerbated microglial responses, DAM-like transcriptomic signatures, and cognitive impairments in female P301S mice but not in males^20^. In contrast, deletion of DAP12 (*Tyrobp*), the adapter protein of Trem2, attenuates neuroinflammation despite exacerbating Tau pathology in female homozygous P301S mice^19^. Consistent with these reports, our observation that *Trem2* deletion prevents DHT-induced exacerbation of neuroinflammation, and Tau pathology corroborates and emphasizes the critical role of Trem2 signaling in mediating female-specific AD pathogenesis.

Our findings also reveal that DHT promotes microglial activation through a mechanism independent of the canonical AR. Although DHT-induced ectopic AR activation in microglia has been implicated in tissue regeneration in multiple sclerosis (MS) models of females^54^, our microglia-specific AR depletion in female OHSCs demonstrates that microglial AR is dispensable for the DHT-induced exacerbation of Tau pathology. This unexpected finding prompted us to search for alternative androgen-responsive transcriptional regulators, leading to the identification of the orphan nuclear receptor TR4. TR4 has previously been implicated in inflammatory signaling and lipid metabolism, which are closely linked to microglial dysfunction during neurodegeneration^41,43,45,46^. Consistent with this notion, we found that TR4 knockdown abolished DHT-induced inflammatory responses, whereas TR4 overexpression phenocopied DHT by inducing *Trem2* and proinflammatory cytokine expression, demonstrating that TR4 is both necessary and sufficient to mediate DHT effects in microglia.

Although our CETSA experiment suggests that DHT interacts with TR4 in intact cells, the precise molecular and biochemical nature of this interaction remains unresolved. One possibility is that DHT engages TR4 in a ligand-receptor fashion. Alternatively, DHT may modulate TR4 activity indirectly through associated cofactors or receptor complexes, such as AR and the estrogen receptor (ER)^45^. Further biochemical and structural studies are needed to distinguish between these possibilities. More broadly, our findings suggest that distinct androgenic and estrogenic signaling pathways converge on TR4 to regulate microglial function. Interestingly, a previous study using aromatase-deficient mice reported exacerbated amyloid pathology through impaired microglial function that could not be recapitulated by ovariectomy alone, suggesting that local steroid metabolism, rather than circulating estrogen levels per se, may play an important role in AD pathogenesis^22^. Future studies using additional hormonal paradigms and looking at different locally relevant signaling pathways are warranted.

Importantly, our study identifies TR4 as a previously unrecognized transcriptional regulator of *Trem2* in microglia. Using luciferase reporter and ChIP-qPCR assays performed both *in vitro* and *in vivo*, we demonstrated that TR4 directly binds the *Trem2* promoter and enhances its transcriptional activity. Interestingly, TR4 is known to transcriptionally regulate ApoE^47^, a Trem2 ligand and a key contributor to Tau-mediated neurodegeneration^13,14,55^, suggesting that TR4 coordinates multiple components of the Trem2 signaling network. Together with our observation that TR4 protein levels are elevated in female human AD brains and correlate with Tau pathology, our data position TR4 as a novel and clinically relevant transcriptional regulator in microglia within the context of female tauopathy. The role of TR4 in microglial biology has only recently begun to emerge, including its involvement in postnatal microglial transition to a heightened vigilance state^56^. Our work extends these observations by identifying TR4 as an upstream regulator of disease-associated microglial activation and identifying a previously unrecognized transcriptional mechanism linking androgen signaling to Tau pathogenesis^57,58^. More broadly, these findings highlight transcriptional control of microglial state transitions as a potentially important mechanism underlying sex-biased neurodegeneration.

## Conclusion

Overall, our findings identify a non-canonical androgen-signaling axis in which the orphan nuclear receptor TR4 transcriptionally activates *Trem2* to drive disease-associated microglial activation and accelerate Tau pathology in female models of tauopathy. The TR4-TREM2 axis serves as a new molecular link between androgen signaling and microglial dysfunction, providing a mechanistic framework for understanding how hormonal dysregulation may contribute to female vulnerability in AD.

## Methods

### Human Samples

Hippocampal and entorhinal cortex tissue from AD female patients in Braak stages 0-II and Braak stage VI was obtained from the UCSD Shiley-Marcos Alzheimer’s Disease Research Center (ADRC). Patient sample details can be found in Supplementary Table 1.

### Mice

Mice were housed in a pathogen-free facility with a 12/12-h light/dark cycle, at a temperature of 68–72°F, and ad libitum access to food and water. To study female vulnerability, we only used female mice in this study. Mice were assigned to age-matched treatment groups in a randomized manner for all experiments. All mice were socially housed within their treatment group.

All mice were sacrificed and tissue harvested in a two-hour time window of 10:00 h to 12:00 h. On the day of sacrifice, mice were anesthetized with a ketamine (100 mg/kg)/xylazine (10 mg/kg) cocktail and transcardially perfused with PBS pH 7.4. After perfusion, tissue was snap frozen or fixed according to the experimental paradigm (for details on OHSCs, see the “Organotypic Hippocampal Slice Cultures (OHSCs)” section). All animal procedures were carried out under the guidelines approved by the Institutional Animal Care and Use Committee of the University of California, San Diego.

For *in vivo* experiments, we used primarily the P301S, also known as PS19, mouse model of tauopathy (B6;C3-Tg(Prnp-MAPT*P301S)PS19Vle/J, Jackson Labs, 008169), which expresses human mutant Tau^1N4R−P301S^ under the direction of the mouse *Prnp* promoter, as previously established^27^. For mouse genotyping, genomic DNA was isolated using the AccuStart II PCR Genotyping Kit and amplified by PCR with AccuStart II GelTrack PCR SuperMix (Quantabio, 95135, 95136). Primers used for P301S genotyping are Forward (WT and hTau+): 5′ TTG AAG TTG GGT TAT CAA TTT GG 3′, Reverse (WT): 5′ TTC TTG GAA CAC AAA CCA TTT C 3′, Reverse (hTau+): 5′ AAA TTC CTC AGC AAC TGT GGT 3′. To generate P301S/Trem2KO mice, we acquired *Trem2*KO mice from The Jackson Laboratory (C57BL/6J-Trem2^em2Adiuj^/J, 027197) and crossed them with the P301S mouse line. P301S/Trem2KO mice were genotyped during crossing until achieving homozygosity using the following primers; Forward (Common): 5’ TCA GGG AGT CAG TCA TTA ACC A 3’, Reverse (WT): 5’ AGT GCT TCA AGG CGT CAT AAG T 3’ and Reverse (Mutant): 5’ CAA TAA GAC CTG GCA CAA GGA 3’.

For *ex vivo* experiments, we used C57BL/6J mice (in-house colony) and *Trem2*KO mice previously mentioned. To generate microglial conditional knockout mice, we crossed an AR^fl/fl^ mouse line carrying flanking loxP sites in exon 2 of the AR (*AR*^*fl/fl*^)^59^ (kindly provided by Dr. Pamela Mellon) with a tamoxifen-inducible Cre line under the Cx3cr1 promoter acquired from The Jackson Laboratory (B6.129P2(C)-Cx3cr1^tm2.1(cre/ERT)Jung^/J, 020940). Primers used for Cx3cr1 Cre genotyping are Forward (WT): 5′ AGC TCA CGA CT GCC TTC TTC 3′, Reverse (Common): 5′ ACG CCC AGA CTA ATG GTG AC 3′, and Forward (Mutant): 5’ GTT AAT GAC CTG CAG CCA AG 3’. Primers used for AR^fl/fl^ genotyping are Forward: 5′ GTT GAT ACC TTA ACC TCT GC 3′ and Reverse: 5′ TTC AGC GGC TCT TTT GAA G 3′. Primers used to evaluate successful conditional knockout are Forward: 5’ GTT GAT ACC TTA ACC TCT GC 3′ and Reverse: 5’ CCT ACA TGT ACT GTG AGA GG 3’.

### Subcutaneous DHT Implant

Implants were prepared as previously described^35^. Briefly, at 6–6.5 months old, ovary-intact female P301S mice were given a subcutaneous implant made of SILASTIC tubing (inner diameter 1.46 mm, outer diameter 1.96 mm) containing 10 mm of DHT powder (Sigma, D-073–1ML) or no DHT (void). Each end of the implants was sealed with ~ 3 mm of silicone adhesive. DHT implants were soaked in sterile physiological saline for 6 h before implantation. Implants were replaced every 4 weeks until the animals were sacrificed at 9–9.5 mo. DHT implants of the same dose completely rescue seminal vesicle weight (an androgen-sensitive measure) and body weight loss in male mice that were gonadectomized (GDX)^35^.

### Stereotaxic injection

Stereotaxic injection of tau fibrils was performed as previously described^38,60^. Briefly, 4-month-old P301S and P301S/TREM2KO mice were anesthetized with inhalation of 2% isofluorane for the duration of surgery and secured on a stereotaxic frame (Kopf Instruments). Mice were then injected stereotaxically at a rate of 0.5 μl/min, with 4 μL of 2 mg/ml K18 PFF into the CA1 region of the left hippocampus. The coordinates for injection were anterior-posterior − 2.5, medial-lateral + 2.0, dorsal-ventral − 1.8. Mice were then implanted with either a void or a DHT-filled implant and sacrificed 4 weeks after injection and transcardially perfused with PBS for immunohistochemistry analysis of Tau aggregation (MC1 + aggregates) in the ipsilateral side. Contralateral side analysis was not performed since little to no aggregation was evident at this time point.

### Immunofluorescence

Cryoprotected mouse brain slices (30 μm in thickness) were placed into wells of 24-well plates and gently washed with sterile 1x PBS 6 times for 10 minutes each time. Tissues were blocked with 5% BSA in 0.4% PBST at room temperature for 1 h and incubated with primary antibodies in 0.4% PBST at 4°C for 48 hours. Antibodies and dilutions were as follows: MC1 (Gift from Peter Davies, distributed via the Feinstein Institute for Medical Research, 1:1000); Iba1(FUJIFILM Wako Chemical, 19741, 1:1000); CD68 (Bio-Rad Laboratories, MCA1957GA,1:500). After 6 times of PBS wash, tissues were incubated with fluorescently labeled secondary antibodies diluted in 0.4% PBST at room temperature for 1 h, followed by 3x PBS washes. The tissues were then mounted on glass slides and coverslipped with Fluoromount-G Mounting Medium (Thermo Fisher Scientific, 00-4958-02). At least three sections corresponding to approximately Bregma − 2.1 to −2.7 were selected, analyzed, and averaged. All mouse brain section images were acquired using an Olympus VS200 slide scanner.

### Morphological analysis of microglia

To analyze microglia 3D morphology, confocal z-stacks were taken using a Nikon AXR confocal system with 12 focal planes of 2-μm intervals. Three fields per mouse of the hippocampal dentate gyrus (DG) were captured. The 3D structure of microglia was reconstructed using the surfaces function in Imaris software, and the filament function was used to determine filament length and branch number.

### DAB staining

Diaminobenzidine (DAB) immunohistochemistry was performed as described above with minor modifications. Briefly, following primary antibody incubation with AT8 (Invitrogen, MN1020; 1:500), sections were washed thoroughly in PBS and incubated with a biotinylated secondary antibody (Invitrogen, anti-Mouse 31800; 1:600) for 1 h, followed by PBS washes and incubation with an avidin-biotin complex (ABC-HRP; Vectastain^®^ Elite ABC-HRP Kit, Vector Laboratories, PK-6100) prepared in 0.4% PBST for 45 minutes before the chromogenic reaction. After PBS washes, sections were incubated with DAB substrate solution prepared with SIGMAFAST DAB Tablets (3,3′-diaminobenzidine, SIGMAFAST^™^, D4293–5SET) according to the manufacturer’s instructions. The chromogenic reaction was monitored visually and allowed to proceed for approximately 5–7 minutes until a brown/black precipitate developed at sites of antigen localization, after which the reaction was terminated by rinsing sections in sodium acetate buffer. Following chromogenic development, sections were mounted onto slides and allowed to dry for a minimum of 24 h. Sections were then dehydrated through a graded ethanol series, cleared in xylene, and coverslipped using DPX mounting medium (Sigma-Aldrich, 06522–100ML). All mouse brain section images were acquired using an Olympus VS200 slide scanner.

### Nissl staining and DG area analysis

Mice hemibrains were cut at 30 μm coronally; all sections containing the hippocampus were collected. Brain sections were mounted on microscope slides (Fisher Scientific) in an anterior-to-posterior order, starting from the section where the hippocampal structure first becomes visible (first section) to the section where the hippocampal structure is no longer discernible (last section). Mice with missing sections were excluded from the analyses, a pre-established criterion. Mounted brain sections were dried at room temperature for at least 24 h at 37°C and cooled down before proceeding with staining. After rehydrating with a brief wash in distilled water, sections were stained in FD Cresyl Violet Solution^™^ (FD NeuroTechnologies, Inc., PS102–1) for 3 minutes. Next, sections were dehydrated in increasing ethanol concentrations and differentiated in 95% ethanol with 0.1% glacial acetic acid. After the final dehydration in 100% ethanol, sections were cleared in xylene and mounted with DPX mounting media (Sigma-Aldrich, 06522–100ML). For DG area analysis, slices at coordinates − 2.2, −2.5, and − 2.7 from Bregma were analyzed by outlining the DG area and measuring the area percentage with the ImageJ function.

### Immunoblotting

For tissue other than OHSCs, mouse and human brain tissue were homogenized in RIPA buffer (150 mM NaCl, 50 mM Tris, 0.5% sodium deoxycholate, 1% Triton X-100, 0.1% SDS, 5 mM EDTA, 20 mM NaF, protease and phosphatase inhibitors: 20 mM NaF, 1:100 dilution 1x protease inhibitor cocktail (PI; Sigma, P8340), 1x phosphatase inhibitor cocktail 2 (PIC2; Sigma, P5726), 1x phosphatase inhibitor cocktail 3 (PIC3; Sigma, P0044), 1 mM PMSF). Mouse tissues were sonicated for 16 pulses. Human tissues were not sonicated. Tissues were centrifuged at 16,000 × g at 4°C for 20 minutes. Supernatants were collected as the RIPA-only fraction, and protein concentrations were determined by the BCA assay (Thermo Fisher Scientific, 23225). Equal amounts of protein were resolved on a 4–12% SDS-PAGE gel (Bio-Rad Laboratories), transferred to a PVDF membrane, and probed with appropriate antibodies. Bands in immunoblots were detected using an enhanced chemiluminescence kit (ECL) (Thermo Fisher Scientific) and visualized using a ChemiDocMP Imaging System (Bio-Rad Laboratories). Band intensity was quantified using ImageLab software (Bio-Rad Laboratories). Total protein was visualized by Imperial (LifeTech, 24615) stain. Representative blots from the same gel/membrane are shown. Antibodies and dilutions used were as follows: Total-Tau (DAKO, A0024; 1:10,000), PHF1 (Gift from Peter Davies, distributed via the Feinstein Institute for Medical Research; 1:1000), AT8 (Invitrogen, MN1020; 1:1000), HT7 (Invitrogen, MN1000; 1:1000), AR(Abcam, ab133273, 1:500); TR4(Abclonal, A6244; 1:500); synaptophysin (Abcam, AB8049,1:1000); CD68 (Bio-Rad Laboratories, MCA1957GA,1:500); actin (Millipore, A2066; 1:2000), GAPDH (CST, 2118; 1:2000), and goat HRP-conjugated secondaries (Invitrogen, anti-Rb 31460, anti-Ms 31430, anti-Rat 31470).

### Reverse Transcriptase and Reverse Transcriptase Quantitative PCR (RT-qPCR)

Total RNA from mouse brains or cell cultures was extracted using the RNeasy Mini Kit (QIAGEN, 74104) following the manufacturer’s protocol. The extracted RNA was subjected to DNase I treatment to eliminate genomic DNA contamination. 500 ng of RNA was reverse transcribed to cDNA using iScript Reverse Transcription Supermix (Bio-Rad Laboratories, 1708841). RT-qPCR was performed using Maxima SYBR Green qPCR Master Mix (Thermo Fisher Scientific, K0253) in the CFX96 Real-Time PCR Detection System (Bio-Rad Laboratories). The expression levels of genes of interest were normalized to that of the housekeeping gene (ACTB), and relative expression fold change was calculated using the 2 − ΔΔCt formula.

Endpoint-PCR to evaluate AR expression in BV2 cells was done using the same reagents as mouse genotyping, using in-house designed primers (Forward: 5’ CAA CCA GAT TCC TTT GCT GCC 3’ and Reverse: 5’ GAG CTT GGT GAG CTG GTA GAA 3’).

### Bulk RNAseq Analysis

Total RNA was isolated from hippocampi of 9–9.5 mo P301S mice using the RNeasy kit (Qiagen). RNA quantity was quantified by spectrophotometer, and integrity was assessed by TapeStation (Agilent). Complementary DNA libraries were prepared from 500 ng of total RNA using the mRNA HyperPrep Kit (KAPA) according to the manufacturer’s recommendations with Unique Dual-Indexed adapters (KAPA). Libraries were PCR-amplified for 10 cycles, and quality was assessed by TapeStation. Libraries were then quantified by Qubit 2.0 fluorometer (Thermo Fisher Scientific), pooled, and analyzed by paired-end 100 bp sequencing on the NovaSeq 6000 platform (Illumina) at UCSD IGM Core. RNA samples were first assessed for sequencing quality, analysis, trimming, and filtering using HTStream (v1.3). Reads were mapped to the mm10 mouse reference genome using STAR (v2.7.10b), and raw transcript counts were generated. Raw counts were imported to R, and differential expression was performed using DESeq2 (v1.42.0). Differential gene expression was represented as a volcano plot, and a cutoff for significance of log2 fold-change > +/− 1 and p-value < 0.05 was used. Gene set enrichment analysis (GSEA) was performed using clusterProfiler gseGO^61,62^ Gene lists were ranked by a score: −logP*FC. The clusterProfiler simplify method was used to reduce the redundancy of enriched GO terms.

### ChIP-qPCR in vivo

In vivo ChIP was performed according to the published report with modifications^52^. Cortex from 9-month-old DHT or void-treated P301S female mice was used for in vivo chromatin immunoprecipitation (ChIP). Briefly, 50–100 mg of frozen cortex was minced and homogenized in ice-cold sucrose buffer, and nuclei were collected by centrifugation. The nuclear fraction was then cross-linked with 1% formaldehyde for 30 minutes at room temperature, quenched with glycine, and washed in cold PBS. Cross-linked nuclei were lysed in DNAzol (Thermo Fisher Scientific, 10503027), and DNA–protein complexes were precipitated with ethanol, washed, and treated under denaturing conditions (SDS/urea) to remove non-covalently bound proteins. The purified chromatin was reprecipitated, resuspended in IP buffer containing SDS and protease inhibitors, and sonicated to obtain sheared chromatin. After confirming fragment size and concentration in 1.5% agarose gel, equal amounts of chromatin (~ 30 μg DNA) were incubated overnight at 4°C with specific antibodies, along with IgG controls. Immune complexes were captured using Protein G magnetic beads, washed thoroughly to reduce background, and eluted. Cross-links were then reversed, samples were treated with proteinase K, and DNA was purified using Monarch^®^ Spin PCR & DNA Cleanup Kit (TS1130S). The recovered DNA was analyzed by qPCR to assess enrichment at target loci relative to input controls. The primers used are Forward: 5’ GTGACATGCCAGCCTCCTAAG 3’ and Reverse: 5’ GGTTATTCTATCTCCTTGCAG 3’.

### Organotypic Hippocampal Slice Cultures (OHSCs)

Organotypic hippocampal slice cultures (OHSCs) were derived from pups of different mouse lines on postnatal days 8–10. Pathological markers were evaluated as described previously with modifications^36–38^ Specifically, female pups were culled by decapitation, and bilateral dissection of the hippocampus was performed in oxygenated artificial cerebrospinal fluid (ACSF): 125 mM NaCl, 2.4 mM KCl, 1.2 mM NaH_2_PO_4_, 1 mM CaCl_2_, 2 mM MgCl_2_, 25 mM NaHCO_3_, and 25 mM Glucose. Approximately 18 to 22 400-μm-thick slices were generated using a tissue chopper, and these slices were cultured in 30 mm Millicell culture inserts (Millipore, PICM0RG50) in 6-well plates (4–5 slices per insert) in OHSCs media: 50% (v/v) Basal Medium Eagle (BME; Thermo Fisher Scientific, 21010046), 25% (v/v) HeatInactivated Horse Serum (Thermo Fisher Scientific, 26050088), 1% (v/v) GlutaMAX (Thermo Fisher Scientific, 35050–061), 0.5% (v/v) Penicillin/Streptomycin (P/S) (Thermo Fisher Scientific, 15070–063), 0.033% (v/v) insulin (Millipore, I9278), 45 mM D-Glucose and 25 mM HEPES (Cytiva, SH30237.01) in EBSS (Thermo Fisher Scientific, 14155063) buffer and sterile-filtered (0.2 μm).

OHSCs were incubated with AAV2-P301ShTau (Virovek) and Tau PFFs (K18/PL) for 24 hours each, followed by treatment with DHT at a concentration of 100 μM or with an equal volume of pure ethanol as a vehicle with every media change according to the experimental paradigm for 14 days. To induce Cre-mediated recombination, 4-hydroxytamoxifen (Sigma-Aldrich, SML1666) was added at a concentration of 5 μg/mL, as previously reported for conditional KO primary microglia culture^40^, during the incubation with AAV and PFFs. Tamoxifen was then removed immediately before starting DHT treatment on DIV 3. Culture media was changed every 2–3 days, and the slices were harvested after 14 days.

### Immunofluorescence for OHSCs

Organotypic hippocampal slices were fixed in 4% paraformaldehyde (PFA) at room temperature for 2–3 h. Following fixation, slices were permeabilized overnight in 1% Triton X-100 in PBS. Antigen retrieval was performed using a citrate buffer (pH 6.0) under low pressure in a pressure cooker for 15 minutes. Slices were then blocked in 20% BSA with 0.4% Triton X-100 in PBS for 3 h at room temperature. Slices were incubated with primary antibodies in 0.4% Triton X-100 in PBS for 48 h at 4°C. Afterward, they were washed six times for 10 minutes each in PBS, followed by incubation with fluorophore-conjugated secondary antibodies and DAPI for 2 h at room temperature. Finally, slices were washed an additional 6 times for 10 minutes in PBS, mounted together with their membrane inserts onto glass slides, and coverslipped using Fluoromount-G Mounting Medium (Thermo Fisher Scientific; 00-4958-02). Images for quantification were acquired using an Olympus IX81 fluorescent microscope using SlideBook software at a magnification of 4x with consistent exposure settings. Percent area quantification was performed using Fiji (ImageJ) with consistent brightness and contrast settings, and thresholding was performed by an experimenter blinded to genotype and treatment. Each translucent dot represents an individual slice and each solid dot represents the average of a single well with 3–5 slices. Images used for figure presentation were taken in a Keyence BXZ-700 at 2x magnification.

### Measurement of Tau species in OHSCs

To measure the levels of soluble and insoluble Tau species, the OHSCs media was removed, washed once with PBS, and placed on ice. Slices were then gently detached from the insert with ice-cold PBS using a pipette and collected in 1.5 mL Eppendorf tubes. Slices were centrifuged at 7,000 × g for a minute, supernatant removed and slices were resuspended with 10 μL of RIPA buffer per slice (150 mM NaCl, 50 mM Tris, 0.5% sodium deoxycholate, 1% Triton X-100, 0.1% SDS, 5 mM EDTA, 20 mM NaF, 1x protease inhibitor cocktail (PI; Sigma, P8340), 1x phosphatase inhibitor cocktail 2 (PIC2; Sigma, P5726), 1x phosphatase inhibitor cocktail 3 (PIC3; Sigma, P0044), pH 8.0). Samples were then homogenized by sonication (Branson Sonifier 250, 5 pulses) and centrifuged at 16,000 × g for 20 minutes. Supernatant was collected as the RIPA-soluble fraction for downstream analysis.

For insoluble species evaluation, the insoluble pellet after centrifugation was resuspended in 20 μL of 2x Laemmli buffer (BioRad; 1610737) with reducing reagent and homogenized by 3 pulses of sonication. This was considered the RIPA insoluble fraction. For immunoblot analysis, the volume of insoluble fraction loaded per lane was normalized to the total protein content of the corresponding RIPA-soluble fraction.

### BV2 cell culture

BV2 cells were cultured in a 10-cm^2^ tissue-treated flask with RPMI 1640 (Corning, 10–040-CV) + 10% Fetal Bovine Serum (FBS; Omega Scientific, FB-02) + 1% antibiotics P/S media (complete RPMI) in a cell culture incubator with 5% CO_2_ at 37°C. When most of the BV2 cells were attached to the bottom of the flask and became 90% confluent, cells were plated at 500,000 per well in 12-well plates and 250,000 on coverslips in 24-well plates for downstream analysis.

### Neuronal conditioned media (NCM) treatment experiment in BV2 cells

NCM treatment was performed as described previously^41^ with minor modifications. Briefly, NCM was collected after five weeks of neuronal differentiation. BV2 cells were seeded onto coverslips as described above, and 24 h after seeding, the cells were treated with 50% NCM from V337M iPSC-neurons and 50% of their original media for 72 h. This was followed by a full media change with either vehicle (DMSO) or 10 nM DHT (Sigma, D-073–1ML) for an additional 24 h. iPSC-derived neurons were maintained as reported previously^41^. Subsequent analysis of RT-qPCR or imaging analysis was performed.

### Tau Inclusion Assay

BV2 cells were plated on coverslips in 24-well plates at 200,000 cells per well in complete RPMI. 24 h after plating, the cells were treated with the culture media containing 50% RPMI 1640 (Corning, 10–040-CV) with 5% FBS. Full-length 2N4R human Tau fibril tagged with ATTO 488 (StressMarq Biosciences Inc., SPR-329-A488) was added at a 2.5 μg/ml concentration, simultaneously with 10 nM DHT (Sigma, D-073–1ML) or DMSO. After 24 hours of incubation, fibrils and drug/hormone were removed, followed by 5 minutes of 0.01% trypsinization to wash off the externally attached fibril. BV2 cells were then fixed in 4% PFA for the acquisition of fluorescent images.

### Immunocytochemistry (ICC)

Cells grown on coverslips in 24-well plates were fixed with 4% PFA at room temperature for 30 minutes, followed by a gentle rinse with cold PBS for 3 times. Cells were permeabilized in 0.1% PBST for 30 minutes, followed by blocking with 2% BSA in 0.02% PBST at room temperature for 1 h. Cells were incubated with primary antibody Iba1(FUJIFILM Wako Chemical, 19741, 1:000) diluted in 2% BSA (in 0.02% PBST) at 4°C overnight without shaking. The next day, cells were washed 3 times in 0.02% PBST for 30 minutes with gentle shaking. Cells were incubated with secondary antibodies diluted in 2% BSA (in 0.02% PBST) at room temperature for 1 h, followed by 3 times 0.02% PBST washes with gentle shaking. The stained cells were stored in PBS at 4°C until mounting with Fluoromount-G Mounting Medium (Thermo Fisher Scientific, 00-4958-02) for imaging. Images were acquired with a Nikon AXR Eclipse Ti2-E confocal system, using a 20× or 60× oil-immersion objective. 488 nm and 568 nm lasers were used to excite GFP and RFP, respectively. Images were taken with the same confocal settings; minor image adjustment (brightness and/or contrast) was performed in ImageJ with the same settings across all the images. For lipid droplet staining, *Bodipy* 493/503 (Invitrogen, D3922) was used in BV2 cell culture in a 1:1000 dilution in PBS.

### CRISPR knockout (KO) in BV2 cells

To generate our *Trem2* KO BV2 line, PX330-GFP vectors encoding single-guide RNAs (sgRNAs) targeting *Trem2* Forward: 5’ TCCCAAGCCCTCAACACCA 3’ and Reverse: 5’ CGTGTGTGCTCACCACACGC 3’ or non-targeting sgRNAs were electroporated into WT BV2 cells using Gene Pulser Xcell Electroporation (Bio-Rad Laboratories). Electroporated cells were recovered for 2 days at 37°C, 5% CO_2_. GFP-positive cells were then sorted via Fluorescence-Activated Cell Sorting (FACS) and kept in complete RPMI for 5 days, during which transiently expressed GFP was diluted out as cells divided. BV2 cells were then stained with APC (Allophycocyanin)-conjugated anti-Trem2 antibody (R&D, FAB17291A), and Trem2 and GFP double-negative cells were sorted as the *Trem2*KO line.

### Generation of TR4 overexpressing BV2

To generate plasmid pLX-EF1a-NR2C2, the human NR2C2 insert was amplified from TFORF2963 (Addgene, 144439) with a 20 bp flanking sequence homologous to pLX-EF1a empty vector (Addgene, 221480). The insert and linearized backbone were ligated by Gibson Assembly. Recombinant plasmids were propagated in *Escherichia coli*, and plasmid sequences were confirmed by Sanger sequencing. To set up transduction, HEK293T cells were cultured in coordination with WT BV2. When HEK cells reached 70% confluence, the plasmid with human *NR2C2* insertion was transfected along with the virus packaging plasmid into HEK cells using PEI. Media containing the virus was collected 72 h after transfection and was centrifuged at 500 g for 5 minutes to pellet any cell debris. The viral supernatant can be aliquoted and stored at −80°C. For BV2 transduction, BV2 cells were pre-seeded 1 day in advance in 12-well plates at 1 × 10^5^ cells per well. 1 ml of lentivirus was mixed with 1 μL 1000X polybrene and then added to 1 × 10^5^ pre-seeded BV2 cells. The cells were spun at 800 g at 32°C for 60 minutes for higher transfection efficiency. TR4 overexpression was confirmed after 3 days.

### siRNA knockdown of TR4

ON-TARGET plus SMARTpool siRNA targeting murine *TR4* (*Nr2c2)* (L-056880-01-0005) and ON-TARGET plus non-targeting control siRNA (D-001810-10-05) were purchased from Dharmacon. BV2 microglial cells were seeded in 6-well tissue culture plates at a density of approximately 1 × 10^6^ cells per well 24 h before transfection and maintained at 37°C in a humidified incubator with 5% CO_2_. On the day of transfection, the culture medium was replaced with 0.75 mL of Opti-MEM (Thermo Fisher Scientific, 31985070) reduced-serum medium. siRNA was used at a final concentration of 25 nM and was mixed with 5 μL of Lipofectamine RNAiMAX (Thermo Fisher Scientific, 13778030) in 250 μL of Opti-MEM (Thermo Fisher Scientific, 31985070). Complexes were incubated for 30 minutes at room temperature before being added dropwise to each well. Cells were incubated with the transfection mixture for 24 h. Following transfection, the medium was replaced with fresh culture medium. Cells were then treated with 15 nM DHT (Sigma, D-073–1ML) and PFFs (2 μg/mL). Vehicle control cells received an equivalent volume of DMSO and PFFs. Cells were incubated for an additional 40 h. Total RNA was extracted using the RNeasy Mini Kit according to the manufacturer’s instructions. cDNA was synthesized using iScript Reverse Transcription Supermix (Bio-Rad Laboratories, 1708841). RT-qPCR was performed using Maxima SYBR Green qPCR Master Mix (Thermo Fisher Scientific, K0253).

### HEK cell culture

HEK293T cells were cultured in Dulbecco’s Modified Eagle Medium (Thermo Fisher Scientific, 11965–092) supplemented with 10% FBS and 1% Penicillin–Streptomycin. Cells were maintained at 37°C in a humidified incubator with 5% CO_2_ and passed every 2–3 days to maintain sub-confluent cultures. Cell density and viability were determined using a hemocytometer following staining with Trypan Blue when required for downstream experiments.

### Motif enrichment analysis and identification of the TR4 binding motif in the *Trem2* promoter

Motif analysis was performed using the SeqPos motif discovery tool (version 0.590) implemented in Galaxy Cistrome, with all annotated motifs in the *Mus musculus* mm10 reference genome used as the background. LISA analysis was performed using the web-based tool (http://lisa.cistrome.org/). Specifically, promoter regions spanning 1 kb upstream and 1 kb downstream of the transcription start sites (TSSs) of DHT-induced genes (adjusted *P* < 0.05, log_2_ fold change > 1) were used as input for motif analysis. SeqPos analysis generated a position-specific scoring matrix (PSSM) for TR4, included in Supplementary Table 2. For LISA analysis, gene symbols corresponding to these DHT-induced genes were used as input.

To identify potential TR4-binding sites within the human and mouse *TREM2* promoters, we used Biomni AI^49^. Specifically, the PSSM of the TR4 binding motif identified by SeqPos, as described above, was submitted to Biomni together with each promoter sequence separately. Human *TREM2* promoter sequence spanned 1031 bp upstream to 198 bp downstream, and the mouse *Trem2* promoter spanned 101–1,000 bp of the TSS. Predicted TR4 binding sites and their associated *P* values were then obtained.

### Luciferase Reporter Assay

*TREM2* gene expression was quantified using a Dual-Luciferase Reporter Assay System (Promega, E1910). HEK293T cells were seeded in 6-well plates at a density of 500,000 cells per well and transfected when they reached 50% confluency using Lipofectamine 3000 (Thermo Fisher Scientific, L3000008). Cells received a firefly luciferase reporter construct carrying the *TREM2* promoter driving firefly luciferase and a Renilla construct under the CMV promoter, along with a construct to overexpress human TR4 or its empty vector. 24 h after transfection, cell lysates were prepared, and luminescence was measured in a Varioskan Lux Multimode Microplate reader (ThermoFisher) per the manufacturer’s protocol. Raw firefly luminescence values were divided by the corresponding protein concentration for each well to generate normalized reporter activity.

### CETSA Classics

CETSA Classics experiments were performed following a previous report, with adaptations^48^. Briefly, HEK293T cells in 10 cm-dishes were trypsinized and resuspended at a density of 20 × 10^6^ cells/mL in Hank’s Balanced Salt Solution (Thermo Fisher Scientific, 24020–117). Cells were split into separate pools and treated with 100 nM DHT (Sigma, D-073–1ML) in DMSO or an equal volume of DMSO as the vehicle group. Following 1 h incubation under tissue culture conditions, cells were then divided into 8-tube strips, 0.2 mL PCR tubes, and heated at the indicated temperatures for 3 minutes in a C1000 Touch Thermal Cycler (Bio-Rad Laboratories). All samples were supplemented with a protease inhibitor cocktail before lysis by 3x freeze-thaw cycles in liquid nitrogen and a water bath. Samples were clarified by centrifugation at 16,000 x g for 20 minutes. Supernatant was collected and mixed with 4x LDS Sample Buffer (Invitrogen, NP0008) with denaturing reagent for downstream analysis.

### Statistical Analysis and Data Representation

Mouse experiments included three independent cohorts of mice. For P301S mice analysis, we used two cohorts for immunostaining and the other hemibrain for bulk RNA-seq, a second cohort was used for biochemical and ChIP analysis. Littermates were randomized to hormonal intervention. All *ex vivo* and *in vitro* experiments were done on at least two independent multi-well plates per experiment, with the control group represented on each plate for batch normalization. Data were normalized to the control group for the experiment, and this is specified throughout as “fold-change” quantifications.

Each point on a graph represents a biological replicate, and technical replicates were summed to the most meaningful biological level. For mouse experiments, each point represents one individual mouse, and any repeated measurements from the same mouse, e.g., immunofluorescent images, were averaged. For the *in vitro* tau inclusion experiment, each semi-transparent point represents a single cell, and a solid point represents a single well. For *in vitro* lipid droplet experiment, each translucent dot in the bar graph represents the average LD number per cell in each ROI, and each solid dot represents the average LD number per cell in each well. For OHSCs, each translucent dot represents a single slice, and each solid dot represents the average of a single well. Group means are shown, and all error bars represent +/− Standard Error of the Mean (SEM). Graphs were made in GraphPad Prism (v. 10) and R (ver. 4.4.3), and aesthetics were adjusted in Adobe Illustrator (2026). Statistical analysis was primarily performed in Prism. p < 0.05, p < 0.01, p < 0.001, p < 0.0001, and not significant are represented as ^*/#*^, ^*/##*^, ^/###^, **^/####^, and ns or numeric value, respectively, and represent pairwise comparisons unless otherwise specified. Statistical tests and multiple comparisons were decided *a priori* and kept consistent throughout experimental designs. For experiments with just two groups, e.g., void vs. DHT, Welch’s t-test was performed. For two-variable experiments, e.g., (WT vs. *Trem2*KO) x (Vehicle vs. DHT), full-model twoway ANOVA and Šídák’s multiple comparisons tests were performed. Area under the curve analysis was calculated in Prism with default settings, then areas were compared by Welch’s t-test.

A linear mixed model was used for the *in vivo* Tau spreading experiment, OHSCs, and cell culture imaging experiments, in which replicates within the same well/mouse are not independent but are included in the analysis to represent biological variability more accurately. This analysis was performed in R (ver. 4.4.3) with the lme4 (ver. 1.1–37) and emmeans (ver. 1.11.2) packages. For OHSC, lipid droplet and tau inclusion assays, treatment was used as a fixed effect and well/sample as a random intercept; this was followed by pairwise comparisons with Šídák correction. For comparison, *in vivo* Tau spreading assay, tau inclusion, OHSCs from WT and *Trem2*KO experiments, and Cre- and Cre+ treatment, and genotype were included as fixed effects, and well/sample/mouse included as a random intercept. Post hoc comparisons were performed using Šídák correction, and p-values were obtained using Satterthwaite’s approximation.

## Supplementary Material

This is a list of supplementary files associated with this preprint. Click to download.

- SupplementaryTables.pdf
- SupplementaryFigures.pdf

**Supplementary Fig. 1. DHT administration exacerbates Tau pathology in a brain tissue-specific manner. a, b** Immunoblot showing increased AR levels in the cortex of P301S DHT-treated females, quantified in (**b**). n = 7 void, n = 6 DHT. **c**, Schematic of DHT treatment paradigm in female organotypic hippocampal slice cultures (OHSCs) (Created in BioRender). **d, e** MC1 staining in female OHSCs treated with vehicle or 100 μM DHT, showing higher MC1 + aggregate area in DHT-treated slices, quantified in (**e**). n = 11 wells/group from three independent experiments. Each translucent dot represents an individual slice, and each solid dot represents an individual well. Scale bar: 500 μm. **f, g** Immunoblot of p-Tau (PHF1), total Tau (DAKO), and GAPDH in RIPA-soluble lysates of female OHSCs treated with vehicle or 100 μM DHT, quantified in (**g**); n = 8 wells per group, from two independent experiments. **b, g** Values are mean ± SEM. ***p < 0.001; numeric values or ns non-significant by Welch’s t-test. **e** Values are the mean ± SEM of individual wells. *p < 0.05 using linear mixed-effect model (LMM).

**Supplementary Fig. 2. Trem2 deletion attenuates Tau pathology, microglial activation, and inflammation ex vivo**. **a, b** Immunostaining of MC1 + Tau aggregates showing that Trem2KO abolishes DHT-induced increase in OHSCs, quantified in (**b**). n = 10–11 wells/group from four independent experiments. Each translucent dot represents an individual slice, and each solid dot represents an individual well. Scale bar: 500 μm. **c, d** Immunoblot showing reduced CD68 in DHT-treated Trem2KO OHSCs, quantified in (**d**); n = 8–10 for vehicle, n = 7–10 for DHT, from three independent experiments. **e** RT-qPCR showing reduced mRNA levels of *TNFα* in DHT-treated Trem2KO OHSCs, n = 8–11 wells/group from three independent experiments. **b** Values are mean ± SEM of individual wells, **,^##^, p < 0.01, ***,^###^p < 0.001 using a multilevel linear mixed-effect model (LMM). **d, e** Values are mean ± SEM, *, ^#^p < 0.05, **,^##^p < 0.01, ***,^###^p < 0.001, ****,^####^p < 0.0001, ns (non-significant) by 2-way ANOVA with Šídák’s multiple comparisons test; * and ^#^ indicate pairwise comparison between different factors.

**Supplementary Fig. 3: AR depletion in microglia does not prevent DHT-induced Tau pathology exacerbation. a** RT-PCR with AR primers fails to detect AR cDNA in BV2 cells. cDNA from female P301S hippocampus was included as a positive control for cDNA-PCR electrophoresis. **b** Schematic outlining tamoxifen and DHT treatment in *AR*^*fl/fl*^; *CX3CR1Cre*^*ERT2+*^ (Cre+) and *AR*^*fl/fl*^; *CX3CR1Cre*^*ERT2−*^ (Cre-) OHSCs. **c** Genomic DNA (gDNA) PCR electrophoresis showing tamoxifen-induced CX3CR1-Cre AR recombination only in Cre+ OHSCs. **d, e** Immunoblot showing no change in p-Tau (PHF-1) or total Tau (DAKO) in RIPA-insoluble lysates between Cre + and Cre− OHSCs treated with tamoxifen, quantified in (**e**). n = 6 wells per group, from three independent experiments. **f, g** MC1 staining showing no change in DHT-induced Tau pathology exacerbation in Cre + and Cre− OHSCs treated with tamoxifen, quantified in (**g**). n = 6 wells/group from three independent experiments. Each translucent dot represents an individual slice, and each solid dot represents an individual well. Scale bar: 500 μm. **e** Values are mean ± SEM, *p < 0.05, **p < 0.01 from 2-way ANOVA with Šídák’s multiple comparisons test. **g** Values are the mean ± SEM of individual wells. *,^#^p<0.05, ns, non-significant using a multilevel linear mixed-effect model (LMM), * and ^#^ indicate pairwise comparison between different factors.

**Supplementary Fig. 4: DHT interacts with TR4 to regulate Trem2 promoter activity. a** LISA analysis on DHT-upregulated genes, identifying TR4 (NR2C2) as the main transcriptional driver. **b** Normalized counts of known TR4 transcriptionally regulated genes (*Rela*, *Apoe, Nfkb1*) showing higher levels in DHT-supplemented P301S females, n = 5 per group. **c** RT-qPCR showing DHT increased *TR4* expression in NCM^V337M^-treated BV2 cells. n = 6 wells from 2 independent experiments. **d** Immunoblot for HA in BV2 cells transduced with TR4 or empty vector, confirming successful TR4 overexpression. **e** Immunoblot of TR4 in lysates from transfected HEK293T cells with either a TR4 overexpressing or empty vector construct, confirming successful transfection of the TR4OE construct. **f, g** Immunoblot of thermostable in-cell (CETSA) TR4 following heat shock at increasing temperatures in HEK293T cells treated with 100 nM DHT or vehicle. Soluble TR4 levels were normalized to the band intensity at 42.5°C and expressed as a percentage. (**g**) Curves were statistically compared by obtaining the area under the curve (AUC). n = 6 repeats from three independent experiments. **h** Schematic of predicted TR4 binding sites in the *TREM2* promoter, identified by Biomni AI. **i** Immunoblot of TR4 in input, IgG, and TR4-immunoprecipitated lysates confirmed successful TR4 immunoprecipitation in P301S female CTX. **b, c, g** Values are mean ± SEM. *p < 0.05, ***p < 0.001, by Welch’s t-test, (g) Values come from AUC analysis.

## Data Availability

The raw sequencing results and alignments for the RNA-seq study are deposited at GEO under accession code GSE329321. This paper does not report original code. For further information and resources, should be directed to and will be fulfilled by the lead contact, Xu Chen (x1chen@health.ucsd.edu).

## References

[1] Miramontes S (2024) Alzheimer’s disease as a women’s health challenge: a call for action on integrative precision medicine approaches. npj Women s Health 2:17–17 38778871 10.1038/s44294-024-00021-3 PMC11106001

[2] Association A (2025) ALZHEIMER’S DISEASE FACTS AND FIGURES SPECIAL REPORT American Perspectives on Early Detection of Alzheimer’s Disease in the Era of Treatment. (2025)

[3] Oveisgharan S (2018) Sex differences in Alzheimer’s disease and common neuropathologies of aging. Acta Neuropathol 136:887–900 30334074 10.1007/s00401-018-1920-1 PMC6279593

[4] Neddens J (2018) Phosphorylation of different tau sites during progression of Alzheimer’s disease. Acta Neuropathol Commun 6:52–52 29958544 10.1186/s40478-018-0557-6 PMC6027763

[5] Liesinger AM (2018) Sex and age interact to determine clinicopathologic differences in Alzheimer’s disease. Acta Neuropathol 136:873–885 30219939 10.1007/s00401-018-1908-x PMC6280837

[6] Filon J (2016) Gender Differences in Alzheimer’s Disease: Brain Atrophy, Histopathology Burden, and Cognition. J Neuropathology Experimental Neurol 75:748–754 10.1093/jnen/nlw047 PMC7299435 27297671

[7] Lopez-Lee C, Torres ERS, Carling G, Gan L (2024) Mechanisms of sex differences in Alzheimer’s disease. Neuron 112:1208–1221 38402606 10.1016/j.neuron.2024.01.024 PMC11076015

[8] Lopez-Lee C (2024) Tlr7 drives sex differences in age- and Alzheimer’s disease–related demyelination. Science 386 10.1126/science.adk7844 PMC12396121 39607927

[9] Casaletto KB (2022) Sex-specific effects of microglial activation on Alzheimer’s disease proteinopathy in older adults. Brain 145:3536–3545 35869598 10.1093/brain/awac257 PMC10233295

[10] Giorgio J (2025) Variable and interactive effects of Sex, APOE ε4 and TREM2 on the deposition of tau in entorhinal and neocortical regions. Nat Commun 16:5812–5812 40595476 10.1038/s41467-025-60370-8 PMC12214702

[11] Biel D (2025) Female sex is linked to a stronger association between sTREM2 and CSF p-tau in Alzheimer’s disease. EMBO Mol Med 17:235–248 39794447 10.1038/s44321-024-00190-3 PMC11822105

[12] Vila-Castelar C (2024) Sex/gender effects of glial reactivity on preclinical Alzheimer’s disease pathology. Mol Psychiatry 30:1430–1439 39384963 10.1038/s41380-024-02753-9 PMC11919761

[13] Shi Y (2017) ApoE4 markedly exacerbates tau-mediated neurodegeneration in a mouse model of tauopathy. Nature 549:523–527 28959956 10.1038/nature24016 PMC5641217

[14] Shi Y (2019) Microglia drive APOE-dependent neurodegeneration in a tauopathy mouse model. J Exp Med 216:2546–2561 31601677 10.1084/jem.20190980 PMC6829593

[15] Mancuso R (2019) CSF1R inhibitor JNJ-40346527 attenuates microglial proliferation and neurodegeneration in P301S mice. Brain 142:3243–3264 31504240 10.1093/brain/awz241 PMC6794948

[16] Keren-Shaul H (2017) A Unique Microglia Type Associated with Restricting Development of Alzheimer’s Disease. Cell 169:1276 28602351 10.1016/j.cell.2017.05.018

[17] Ulland TK, Colonna M (2018) TREM2 — a key player in microglial biology and Alzheimer disease. Nat Reviews Neurol 14:667–675 10.1038/s41582-018-0072-1 30266932

[18] Leyns CEG (2017) TREM2 deficiency attenuates neuroinflammation and protects against neurodegeneration in a mouse model of tauopathy. Proceedings of the National Academy of Sciences 114, 11524–11529 10.1073/pnas.1710311114 PMC5663386 29073081

[19] Chen H (2025) DAP12 deletion reduces neuronal SLIT2 and demyelination and enhances brain resilience in female tauopathy mice. Mol Neurodegeneration 20:124–124 10.1186/s13024-025-00903-3 PMC12673724 41331787

[20] Sayed FA (2021) AD-linked R47H-TREM2mutation induces disease-enhancing microglial states via AKT hyperactivation. Sci Transl Med 13 10.1126/scitranslmed.abe3947 PMC9345574 34851693

[21] McCarthy M, Raval AP (2020) The peri-menopause in a woman’s life: a systemic inflammatory phase that enables later neurodegenerative disease. J Neuroinflamm 17:317–317 10.1186/s12974-020-01998-9 PMC7585188 33097048

[22] Yue X (2005) Brain estrogen deficiency accelerates Aβ plaque formation in an Alzheimer’s disease animal model. Proceedings of the National Academy of Sciences 102, 19198–19203 10.1073/pnas.0505203102 PMC1323154 16365303

[23] Li R, Cui J, Shen Y (2014) Brain sex matters: Estrogen in cognition and Alzheimer’s disease. Mol Cell Endocrinol 389:13–21 24418360 10.1016/j.mce.2013.12.018 PMC4040318

[24] Muñoz-Mayorga D, Guerra-Araiza C, Torner L, Morales T (2018) Tau Phosphorylation in Female Neurodegeneration: Role of Estrogens, Progesterone, and Prolactin. Front Endocrinol 9:133–133 10.3389/fendo.2018.00133 PMC5882780 29643836

[25] Brinton RD, Yao J, Yin F, Mack WJ, Cadenas E (2015) Perimenopause as a neurological transition state. Nat Reviews Endocrinol 11:393–405 10.1038/nrendo.2015.82 PMC9934205 26007613

[26] Mauvais-Jarvis F, Lindsey SH (2024) Metabolic benefits afforded by estradiol and testosterone in both sexes: clinical considerations. J Clin Invest 134 10.1172/JCI180073 PMC11364390 39225098

[27] Huddleston HG (2024) Associations of Polycystic Ovary Syndrome With Indicators of Brain Health at Midlife in the CARDIA Cohort. Neurology 102 10.1212/WNL.0000000000208104 PMC11383880 38295344

[28] Kauffman AS (2024) Androgen Inhibition of Reproductive Neuroendocrine Function in Females and Transgender Males. Endocrinology 165 10.1210/endocr/bqae113 PMC11393496 39207217

[29] Sarahian N, Sarvazad H, Sajadi E, Rahnejat N, Roozbahani NE (2021) Investigation of common risk factors between polycystic ovary syndrome and Alzheimer’s disease: a narrative review. Reproductive Health 18:156–156 34311759 10.1186/s12978-021-01203-x PMC8314638

[30] Lakshmikanth T (2024) Immune system adaptation during gender-affirming testosterone treatment. Nature 633:155–164 39232147 10.1038/s41586-024-07789-z PMC11374716

[31] Sobczuk J, Paczkowska K, Andrusiów S, Bolanowski M, Daroszewski J (2024) Are Women with Polycystic Ovary Syndrome at Increased Risk of Alzheimer Disease? Lessons from Insulin Resistance, Tryptophan and Gonadotropin Disturbances and Their Link with Amyloid-Beta Aggregation. Biomolecules vol. 14 918–918 39199306 10.3390/biom14080918 PMC11352735

[32] Yoshiyama Y (2007) Synapse Loss and Microglial Activation Precede Tangles in a P301S Tauopathy Mouse Model. Neuron 53:337–351 17270732 10.1016/j.neuron.2007.01.010

[33] Swerdloff RS, Dudley RE, Page ST, Wang C, Salameh W, Dihydrotestosterone (2017) Biochemistry, Physiology, and Clinical Implications of Elevated Blood Levels. Endocr Rev 38:220–254 28472278 10.1210/er.2016-1067 PMC6459338

[34] Mauvais-JarvisF, BhasinS (2026) Metabolic Messengers: testosterone. Nat Metabolism 8:52–6110.1038/s42255-025-01431-641514077

[35] Esparza LA, Terasaka T, Lawson MA, Kauffman AS (2020) Androgen Suppresses In Vivo and In Vitro LH Pulse Secretion and Neural Kiss1 and Tac2 Gene Expression in Female Mice. Endocrinology 161 10.1210/endocr/bqaa191 PMC7671291 33075809

[36] Croft CL, Noble W (2018) Preparation of organotypic brain slice cultures for the study of Alzheimer’s disease. F1000Research 7:592–592 29904599 10.12688/f1000research.14500.1 PMC5964634

[37] Croft CL (2017) Membrane association and release of wild-type and pathological tau from organotypic brain slice cultures. Cell Death Dis 8 10.1038/cddis.2017.97 PMC5386587 28300838

[38] Jati S (2025) Chromogranin A deficiency attenuates tauopathy by altering epinephrine–alpha-adrenergic receptor signaling in PS19 mice. Nat Commun 16:4703–4703 40393970 10.1038/s41467-025-59682-6 PMC12092710

[39] Abdulkhaliq AA (2026) TREM2 in neurodegeneration and diseases. Mol Psychiatry. 10.1038/s41380-026-03505-7 PMC13268972 41792456

[40] Wang C (2022) Microglial NF-κB drives tau spreading and toxicity in a mouse model of tauopathy. Nat Commun 13:1969–1969 35413950 10.1038/s41467-022-29552-6 PMC9005658

[41] Li Y (2024) Microglial lipid droplet accumulation in tauopathy brain is regulated by neuronal AMPK. Cell Metabol 36:1351 10.1016/j.cmet.2024.03.014 PMC11153007 38657612

[42] Yao P (2017) Androgen alleviates neurotoxicity of β-amyloid peptide (Aβ) by promoting microglial clearance of Aβ and inhibiting microglial inflammatory response to Aβ. CNS Neurosci Ther 23:855–865 28941188 10.1111/cns.12757 PMC6492702

[43] Xie S (2009) TR4 nuclear receptor functions as a fatty acid sensor to modulate CD36 expression and foam cell formation. Proceedings of the National Academy of Sciences 106, 13353–13358 10.1073/pnas.0905724106 PMC2726407 19666541

[44] Kang HS (2010) Nuclear Orphan Receptor TAK1/TR4-Deficient Mice Are Protected Against Obesity-Linked Inflammation, Hepatic Steatosis, and Insulin Resistance. Diabetes 60:177–188 20864514 10.2337/db10-0628 PMC3012170

[45] Lin S (2014) Pathophysiological Roles of the TR4 Nuclear Receptor: Lessons Learned From Mice Lacking TR4. Mol Endocrinol 28:805–821 Minireview 24702179 10.1210/me.2013-1422 PMC4042077

[46] Min Z (2023) NR2C2 of macrophages promotes inflammation via NF-κB in LPS-induced orchitis in mice. Reproduction 166:209–220 37427695 10.1530/REP-23-0041

[47] Kim E, Yang Z, Liu N-C, Chang C (2005) Induction of apolipoprotein E expression by TR4 orphan nuclear receptor via 5′ proximal promoter region. Biochem Biophys Res Commun 328:85–90 15670754 10.1016/j.bbrc.2004.12.146

[48] Shaw J (2018) Determining direct binders of the Androgen Receptor using a high-throughput Cellular Thermal Shift Assay. Sci Rep 8:163–163 29317749 10.1038/s41598-017-18650-x PMC5760633

[49] Huang K (2025) Biomni: A General-Purpose Biomedical AI Agent. bioRxiv (Cold Spring Harbor Laboratory) Preprint at 10.1101/2025.05.30.656746

[50] Hu L (2019) Targeting TR4 nuclear receptor with antagonist bexarotene increases docetaxel sensitivity to better suppress the metastatic castration-resistant prostate cancer progression. Oncogene 39:1891–1903 31748715 10.1038/s41388-019-1070-5 PMC7044111

[51] Xia L (2021) Targeting the TR4 nuclear receptor with antagonist bexarotene can suppress the proopiomelanocortin signalling in AtT-20 cells. J Cell Mol Med 25:2404–2417 33491272 10.1111/jcmm.16074 PMC7933964

[52] Li L, Akl MG, Widenmaier SB (2025) Protocol for in vivo chromatin immunoprecipitation on purified chromatin isolated from mouse liver nuclei. STAR Protocols 6:103616–103616 39891912 10.1016/j.xpro.2025.103616 PMC11835655

[53] Ocañas SR (2023) Microglial senescence contributes to female-biased neuroinflammation in the aging mouse hippocampus: implications for Alzheimer’s disease. J Neuroinflamm 20:188–188 10.1186/s12974-023-02870-2 PMC10433617 37587511

[54] Zahaf A (2023) Androgens show sex-dependent differences in myelination in immune and non-immune murine models of CNS demyelination. 10.1038/s41467-023-36846-w PMC10033728 36949062

[55] Krasemann S (2017) The TREM2-APOE Pathway Drives the Transcriptional Phenotype of Dysfunctional Microglia in Neurodegenerative Diseases. Immunity 47:566 28930663 10.1016/j.immuni.2017.08.008 PMC5719893

[56] Han CZ (2023) Human microglia maturation is underpinned by specific gene regulatory networks. Immunity 56:2152 37582369 10.1016/j.immuni.2023.07.016 PMC10529991

[57] Depp C, Doman JL, Hingerl M, Xia J, Stevens B (2025) Microglia transcriptional states and their functional significance: Context drives diversity. Immunity vol. 58 1052–1067 40328255 10.1016/j.immuni.2025.04.009

[58] Ardura-Fabregat A (2025) Response of spatially defined microglia states with distinct chromatin accessibility in a mouse model of Alzheimer’s disease. Nat Neurosci 28:1688–1703 40659845 10.1038/s41593-025-02006-0 PMC12321583

[59] Yeh S (2002) Generation and characterization of androgen receptor knockout (ARKO) mice: An in vivo model for the study of androgen functions in selective tissues. Proceedings of the National Academy of Sciences 99, 13498–13503 10.1073/pnas.212474399 PMC129702 12370412

[60] Chen X (2020) Promoting tau secretion and propagation by hyperactive p300/CBP via autophagy-lysosomal pathway in tauopathy. Mol Neurodegeneration 15:2–2 10.1186/s13024-019-0354-0 PMC6945522 31906970

[61] Mootha VK (2003) PGC-1α-responsive genes involved in oxidative phosphorylation are coordinately downregulated in human diabetes. Nat Genet 34:267–273 12808457 10.1038/ng1180

[62] Subramanian A (2005) Gene set enrichment analysis: A knowledge-based approach for interpreting genome-wide expression profiles. Proceedings of the National Academy of Sciences 102, 15545–15550 10.1073/pnas.0506580102 PMC1239896 16199517

